# Traditional Chinese Medicines as Effective Reversals of Epithelial-Mesenchymal Transition Induced-Metastasis of Colorectal Cancer: Molecular Targets and Mechanisms

**DOI:** 10.3389/fphar.2022.842295

**Published:** 2022-03-04

**Authors:** Hongzhang Ge, Chao Xu, Haitao Chen, Ling Liu, Lei Zhang, Changhong Wu, Yi Lu, Qinghua Yao

**Affiliations:** ^1^ Department of Integrated Traditional Chinese and Western Medicine, The Cancer Hospital of the University of Chinese Academy of Sciences (Zhejiang Cancer Hospital), Institute of Basic Medicine and Cancer (IBMC), Chinese Academy of Sciences, Hangzhou, China; ^2^ Key Laboratory of Integration of Chinese and Western Medicine Oncology, Zhejiang Cancer Hospital, Hangzhou, China; ^3^ Key Laboratory of Head and Neck Cancer Translational Research of Zhejiang Province, Zhejiang Cancer Hospital, Hangzhou, China; ^4^ Second Clinical Medical College, Zhejiang Chinese Medical University, Hangzhou, China; ^5^ Department of Clinical Nutrition, The Cancer Hospital of the University of Chinese Academy of Sciences (Zhejiang Cancer Hospital), Institute of Basic Medicine and Cancer (IBMC), Chinese Academy of Sciences, Hangzhou, China

**Keywords:** colorectal cancer, Traditional Chinese medicines, epithelial-mesenchymal transition, EMT-related signaling pathways, tumor metastasis

## Abstract

Colorectal cancer (CRC) is the third most common type of cancer worldwide. Distant metastasis is the major cause of cancer-related mortality in patients with CRC. Epithelial-mesenchymal transition (EMT) is a critical process triggered during tumor metastasis, which is also the main impetus and the essential access within this duration. Therefore, targeting EMT-related molecular pathways has been considered a novel strategy to explore effective therapeutic agents against metastatic CRC. Traditional Chinese medicines (TCMs) with unique properties multi-target and multi-link that exert their therapeutic efficacies holistically, which could inhibit the invasion and metastasis ability of CRC cells via inhibiting the EMT process by down-regulating transforming growth factor-β (TGF-β)/Smads, PI3K/Akt, NF-κB, Wnt/β-catenin, and Notch signaling pathways. The objective of this review is to summarize and assess the anti-metastatic effect of TCM-originated bioactive compounds and Chinese medicine formulas by mediating EMT-associated signaling pathways in CRC therapy, providing a foundation for further research on the exact mechanisms of action through which TCMs affect EMT transform in CRC.

## Introduction

Colorectal cancer (CRC) is the third most commonly diagnosed malignancy and the second leading cause of cancer-related mortality in the world (Keum and Giovannucci, 2019). There are 1.8 million new cases and mortality of 0.88 million in 2018 worldwide (Bray et al., 2018), and over 376,000 new cases and 191,000 deaths are estimated to occur annually in China (Arnold et al., 2017). Furthermore, the incidence of CRC will reach 2.2 million new cases and 1.1 million deaths by 2030 (Arnold et al., 2017), which will lead to a significant economic burden increase by 60%, and it has become a severe public health problem worldwide.

In recent decades, therapeutic strategies and agents for CRC treatment have made a lot of progress and innovation. However, it is hard to tackle once cancer distant metastases have occurred and spread, ultimately resulting in poorer prognosis and high mortality in patients with CRC (Lambert et al., 2017; Mun et al., 2019). In 20–25% of patients with colonic cancer and 18% of patients with rectal cancer, metastatic cases are present at the time of the first diagnosis (De Rosa et al., 2015; Piawah and Venook, 2019). The liver represents the main metastatic localization account for 30–60%, and the 5-year survival rate after metastasis is only 20% in CRC patients (Mattar et al., 2016; Chapelle et al., 2018). In addition, it is also worried that the majority of recurrences occurred within the first 3 years after surgical resection, and approximately 95% of metastatic cases appear in the first 5 years. Hence, tumor metastasis remains a huge challenge in clinical CRC treatment and management, and there is an urgent need for developing potential and effective therapeutic agents to handle it.

It has been proven that epithelial-mesenchymal transition (EMT) plays a pivotal role in carcinoma metastasis (Lamouille et al., 2014; Bakir et al., 2020). The EMT transformation facilitates converting tumor cells from epithelial cells to mesenchymal ones, leading to the loss of cell polarity and the ability of cell adhesion and acquiring some properties of a mesenchymal phenotype, including heightening cancer cells invasion and metastasis capabilities (Hao et al., 2019). Subsequently, the transformed cancer cells easily penetrate the basement membrane and spread into the lymphatic and blood circulation (Karlsson et al., 2017), and then invade surrounding tissues and migrate to distant organs, primarily including liver and lung. After seeding, these tumor cells transform to an epithelial phenotype and proliferate to form secondary metastatic foci at distant sites (Thiery et al., 2009; Dongre and Weinberg, 2019). Taken together, with the EMT program has been increasingly recognized to triggering tumor metastasis (Diepenbruck and Christofori, 2016; Mittal, 2018), and eventually resulting in obstruction of successful CRC treatment (Erin et al., 2020), suggesting that targeting EMT mediators may represent an effective and reliable strategy for therapeutic intervention of CRC.

Traditional Chinese medicine (TCM) is a type of natural medicine, which is well known for its indispensable role in cancers clinical treatment and has been increasingly used in the past decades worldwide (Ma Z. et al., 2019). In addition, TCMs have been shown to be effective in postponing cancer recurrence and suppressing metastasis (Ma et al., 2017; Wang Y. et al., 2020), and improving the living quality of cancer patients (Wang K. et al., 2021). In the combat against metastatic CRC, accumulating evidence demonstrated that TCM monomer compounds and Chinese medicine formulations based on TCM theory can exert inhibitory effects on invasion and metastasis of cancer cells via targetting EMT-related signaling pathways (Liu et al., 2018; Bai et al., 2019; Luo et al., 2019). Therefore, TCMs as a complementary and alternative medical therapy to fight against the tumor metastasis of CRC has been increasingly accepted, and finding potential therapeutic candidates is very required by targeting EMT process from its abundant resource.

Up to now, a large number of natural bioactive compounds, medicinal herb extracts, and Chinese medicine formulations with variable results have been examined with the purpose of controlling the pathological EMT process. In this review, we summarize the recent researches to analyze and discuss these TCMs exert the regulatory effects on metastatic and invasive characteristics through the crucial molecular mechanisms involved in EMT, expectantly finding more potential therapeutic candidates used for the prevention and treatment of metastatic CRC.

## EMT-Related Signalings Involved in Metastasis of CRC and EMT Reversal Agents

Tumor metastasis (Lambert et al., 2017) is the primary cause of cancer lethality, it is a progressive and multistep dynamic process, including detachment from a primary tumor, invasion into surrounding tissues, diffusion into blood/lymphatic circulation, and extravasation from blood vessels, then distal seeding. The migration of CRC cells plays a crucial role in tumor metastasis, and the changes in CRC cells acquired from EMT transform is a vital factor that promotes the tumor cells to escape from the primary site (Lee and Lotze, 2009).

**FIGURE 1:**
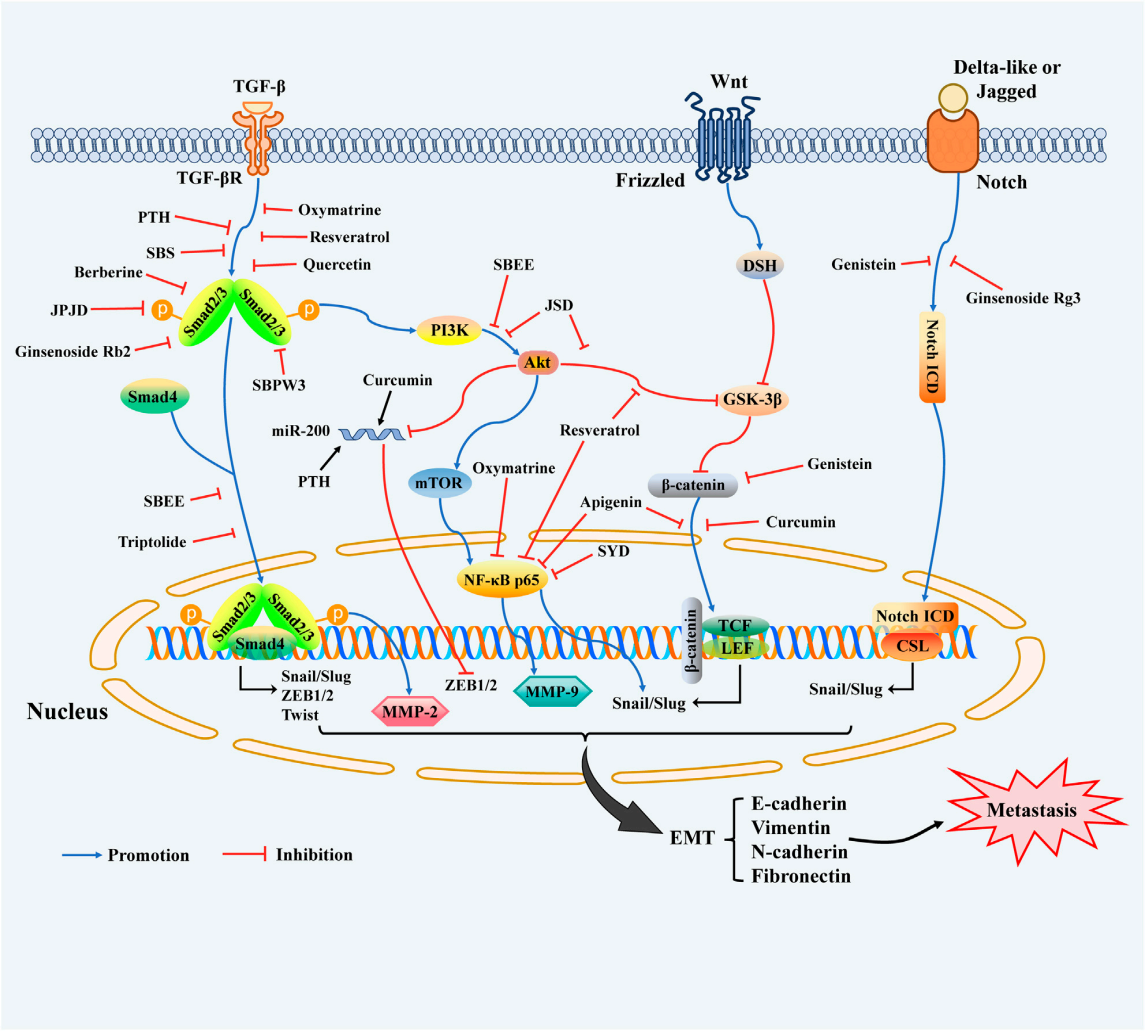
Key molecular targets and related-signaling pathways during the EMT program involved in the inhibition of migratory and invasive characteristics of CRC by TCMs. Notes: Several cell-intrinsic signalings cooperate to induce the expression of EMT-TFs containing Snail, Slug, ZEB1/2, and Twist, the expressions of which are induced by the above-mentioned signaling pathways, including TGF-β/Smads, Notch, Wnt/β-catenin, PI3K/Akt, and NF-κB, ultimately leading to transformation to the mesenchymal cell state. In response to TGF-β, Smad2/3 is activated and forms a protein complex with Smad4, and promotes the transcription of EMT-TFs. the expression of which is induced by various signaling pathways. The TGF-β molecular pathway also collaborates with the PI3K/AKT pathway, which in turn triggers the activation of the mTOR complex and NF-κB. On the other hand, AKT can restrain the function of GSK-3β, a kinase that inhibits nuclear translocation of *ß*-catenin and EMT-TFs Snail and Slug in Smad-independent pathways. Furthermore, MMP-2 expression is positively controlled by Smad2 whilst MMP-9 is modulated via NF-κB p65/p50 transcription signaling in tumor tissues and cells of CRC. The miR-200 family can down-regulate ZEB1/2 expression, and miR-200 can be blocked by Akt, which is triggered by most EMT-associated signalings. Wnt signaling promotes EMT programme by repressing GSK-3β through DSH to stabilize *ß*-catenin, which translocates into the nucleus to engage the transcription factors TCF and LEF and facilitate EMT-related genes expression. Additionally, Notch signaling also participates in EMT progression, behaving as interactions of Delta-like or Jagged ligands with Notch receptors initiate signaling through the proteolytic release of the Notch ICD, and then modulates target gene expression.

Increasing evidence indicated that an important reason for CRC metastasis is the enhanced migration and invasion of cancer cells attributed to EMT phenotypes. EMT is a reversible and dynamic progression that transiently places epithelial cells into mesenchymal cells and affects the cellular architecture, which directly elevates tumor-initiating, invasive, and migratory characteristics (Dongre and Weinberg, 2019; Guo et al., 2021). Recently, numerous researches have confirmed that EMT progression is mainly mediated by multiple key signal transduction pathways containing transforming growth factor-β (TGF-β)/Smads, Wnt/β-catenin, phosphatidylinositol 3-kinase (PI3K)/Akt, Notch, and NF-κB signaling pathways (De Rosa et al., 2015; Vinson et al., 2016; Mishra et al., 2017; Tan et al., 2017; Song et al., 2020). There are multiple specific EMT-transcription factors (EMT-TFs) involved in the EMT process undergoing tumor metastasis, including Snail, Slug (Snai2), ZEB1/2, and Twist (Goossens et al., 2017; Williams et al., 2019; Feng et al., 2020; Kumari et al., 2021; Lu et al., 2021), they closely acted pleiotropically and in diverse combinations to down-regulate epithelial marker E-cadherin, while up-regulating the mesenchymal signatures such as N-cadherin, vimentin, and fibronectin, and thus promoting the tendency of cancer cells to mesenchymal-like features (Dongre and Weinberg, 2019), which ultimately contribute to the initiation of EMT process. The transcriptional modulation of multiple signal pathways with crosstalk molecular mechanisms closely involved in EMT regulation are summarized as indicated in Figure 1.

Taken together, an advancing understanding of investigating the molecular mechanisms involved in regulating EMT transform is highly desirable for the development of the novel specific therapeutic strategy and agent or agent for the treatment of metastatic CRC.

### TGF-β/Smad Signaling in EMT

The TGF-β/Smad signaling plays an essential and heterogeneous role in CRC and is a crucial driver of the EMT (Lampropoulos et al., 2012; David and Massague, 2018; Lin and Wu, 2020). TGF-β is a secreted multifunctional cytokine that regulates a variety of cellular processes. In the case of the canonical Smad-dependent pathway, the TGF-β signaling pathway is activated by binding of TGF-β ligands to type I and type II cell surface receptors (Hao et al., 2019), respectively, and recruits and phosphorylates Smad2 and Smad3 proteins, then allow to form heteromeric complexes combine with Smad4. Then the trimeric Smad heterocomplexes translocate into the nucleus and activate the mesenchymal phenotype genes involve master regulators, including Snail, Slug, Twist, and ZEB1/2 (Lamouille et al., 2014), whereas repressing the epithelial phenotype, thereby acquired the EMT phenotype (Avila-Carrasco et al., 2019), eventually facilitating tumor cell invasion and metastasis in CRC progression.

#### Resveratrol

Resveratrol was first derived from the roots of *Veratrum grandiflorum* (Maxim. ex Miq.) O.Loes. [Melanthiaceae] (Li et al., 2019b; Hou et al., 2019), which is also present in various medicinal plants, such as *Morus rubra* L. [Moraceae], *Reynoutria japonica* Houtt. [Polygonaceae], and *Arachis hypogaea* L. [Fabaceae] (Li et al., 2019b; Hou et al., 2019). It is noteworthy that resveratrol has shown to exert various pharmacological activities, such as anti-oxidative, anti-cardiovascular, anti-inflammatory, and anti-cancer (Kursvietiene et al., 2016; Oh and Shahidi, 2018), and it has been widely used in protecting against atherosclerosis, cardiomyopathy, and various cancers (Borriello, 2017; Cheng et al., 2020).

Recently, some studies have demonstrated that resveratrol has a potential effect on the intervention of tumor migration and invasion. For instance, a pharmacological study demonstrated that resveratrol (Ji et al., 2015) suppressed the hepatic and lung metastases in orthotopic CRC tumor mice model. In addition, resveratrol inhibited EMT progression in CRC LoVo cells via inhibiting the TGF-β1/Smads signaling pathway.

#### Quercetin

Quercetin is a common polyphenol component that widely exists in multiple Chinese herbs, including *Bupleurum falcatum* L. [Apiaceae], *Lygodium japonicum* (Thunb.) Sw. [Schizaeaceae], and *Lysimachia christinae* Hance [Primulaceae] (Darband et al., 2018; Sun et al., 2019). Like several other compounds with a flavone ring, quercetin also exerts a remarkable effect on the immunity and inflammation. In particular, it has been found that quercetin has several pharmacological benefits containing promotion of apoptosis, cell cycle arrest, and inhibition of metastasis (Darband et al., 2018), which is extensively used to inhibit digestive system cancers, including esophageal cancer, pancreatic cancer, and CRC (Pang et al., 2019; Davoodvandi et al., 2020; Lin et al., 2020). Moreover, there is a clinical meta-analysis suggested that intake of a large amount of quercetin may be beneficial in reducing the incidence risk of colon cancer (Chang et al., 2018).

It has revealed that EMT-TF Twist1 is competent at dedifferentiating epithelial type tumor cells into non-proliferative, mobile mesenchymal cells with stemness characteristics (Tsai et al., 2012). One prior study showed that quercetin (Feng et al., 2018) repressed TGF-β1-induced EMT and invasion potential by blocking the inhibitory effect of Twist1 on the E-cadherin promoter in CRC SW480 cells. Reduction of Twist1 can reversibly redifferentiate the EMT cells into epithelial-phenotypes cells at the site of metastasis where the cells start colonizing and proliferating, potentially contributing to inhibiting tumor metastasis and invasion. Additionally, it is demonstrated that quercetin was strongly conducive to reversing the EMT programme by mediating EMT indicators containing *ß*-catenin, E-cadherin, N-cadherin, and EMT-associated transcription factor Snail (Kee et al., 2016).

#### Berberine

Berberine is a natural benzyl tetra isoquinoline alkaloid present in various traditional medicinal herbs, such as *Coptis chinensis* Franch. [Ranunculaceae], *Hydrastis canadensis* L. [Ranunculaceae], *Phellodendron amurense* Rupr. [Rutaceae], and *Berberis vulgaris* L. [Berberidaceae] (Chen H. et al., 2020; Warowicka et al., 2020). Berberine has been widely proven to be effective in treating gastroenteritis patients with diarrhea in China for a long time (Imenshahidi and Hosseinzadeh, 2016). Increasing studies have also displayed that berberine is characterized by multiple pharmacological effects including anti-inflammatory (Kong WJ. et al., 2020), anti-microbial (Yu et al., 2020), anti-viral (Warowicka et al., 2020), and anti-cancer properties (Wang K. et al., 2017). It was reported that berberine significantly suppressed the growth of gastrointestinal cancers cells in multiple preclinical models (Hesari et al., 2018; Zhang Q. et al., 2020).

Of note, a clinical investigation has revealed that berberine has an attenuative efficacy on colorectal adenoma (Chen YX. et al., 2020). One prior literature (Huang et al., 2020) indicated that berberine in a concentration-dependent manner inhibited EMT programme by regulation of the Smad-dependent and Smad-independent TGF-β signaling pathways, acting as markedly decreasing the ZEB1 and Snail expression, and also down-regulating the expressions of TβRI, TβRII, Smad2/p-Smad2, and Smad3/p-Smad3 *in vitro*. Moreover, demethyleneberberine is a structural analogue of berberine, effectively inhibiting the EMT process by inhibiting the TGF-β/Smads signaling pathway in CRC HCT116 cells (Wang Z. et al., 2021).

#### Ginsenoside Rb2

*Panax ginseng* C.A.Mey. [Araliaceae] (ginseng), one of the most appreciated traditional herbal medicines that has been generally used in clinical traditional Chinese medicine practices traced to 5, 000 years ago in China. The widely popular health-promoting effects of ginseng involved in restorative, antioxidant, anti-microbial, anti-ageing, anti-cardiovascular, and anti-cancer properties (Wu et al., 2018; Wang L. et al., 2020; Fan et al., 2020). Currently, more than 50 types of ginsenosides are a group of triterpenoid saponins isolated and identified from ginseng, which have been proposed to account for the primary biological activities of ginseng (Sabouri-Rad et al., 2017).

Ginsenoside Rb2 has attracted increasing attention on anti-CRC in diverse pharmacological researches. Notably, ginsenoside Rb2 was found to decrease colony-forming ability, metastasis, and invasion *in vitro*, and the mesenchymal indicators containing Snail, twist, fibronectin, vimentin, and MMP-2 were markedly down-regulated in the presence of ginsenoside Rb2, whereas E-cadherin was up-regulated in both HT29 and SW620 CRC cell lines (Phi et al., 2018). Moreover, it is noteworthy that ginsenoside Rb2 can bond to the hydrophobic pocket of TGF-β1, resulting in destructing TGF-β1 dimerization by the docking simulation assay. Additionally, it has been confirmed to strongly inhibit tumor metastasis and EMT process of human CRC cells by restraining the TGF-β/Smad signaling pathway in HCT116 and SW620 cells. (Dai et al., 2019).

#### Oxymatrine

Oxymatrine, a natural quinolizidine alkaloid, is the main biological active compound separated from the roots of Chinese herb *Sophora flavescens* Aiton [Fabaceae] (Xia et al., 2011). Recently, accumulating researches have exhibited a wide range of pharmacological efficacies of oxymatrine containing anti-inflammatory, antiviral, immunomodulatory, and cardiovascular protective effects (Cao et al., 2010; Halim et al., 2019; Jin et al., 2020). Additionally, oxymatrine has been revealed to be effective in potential anti-cancer properties via multiple molecular pathways, including suppression of proliferation, induction of apoptosis, and inhibition of metastasis (Chen et al., 2019; Guo and Yang, 2019).

Plasminogen activator inhibitor-1 (PAI-1) is a crucial target gene in the TGF-β/Smad signaling, which can block the degradation of extracellular matrix (ECM) composition and promote the invasive and migratory properties of cancer cells. It is documented that oxymatrine inhibited the migration ability by alleviating EMT transform induced via suppressing the activation of TGF-β1/Smads signaling by repressing PAI-1 expression in CRC RKO cells (Wang X. et al., 2017).

#### *Scutellaria Barbata* D.Don [Lamiaceae] Ethanol Extract

*Scutellaria barbata* D.Don [Lamiaceae] is a promising medicinal herb that is considered to be effective in heat-clearing, detoxification, facilitation of blood circulation, and clearance of blood stasis in the TCM field. It has long been used as a crucial ingredient in several Chinese herbal formulations for the clinical treatment of diverse types of cancers including CRC (Zhang L. et al., 2017). Interestingly, it has been reported that the *Scutellaria barbata* D.Don [Lamiaceae] ethanol extract (SBEE) can promote apoptosis and repress cell proliferation in colon cancer cells (Lin J. et al., 2014; Wei et al., 2017).

A prior study revealed that SBEE exerted inhibitory effects on the migratory and invasive characteristics of HCT-8 cells via repressing the activation of the TGF-β/Smad signaling (Jin et al., 2017). Furthermore, SBPW3 is a homogeneous polysaccharide separated from the plant of *Scutellaria barbata* D.Don [Lamiaceae], which significantly repressed TGF-β1-induced metastasis and invasion of CRC HT-29 cells through reversing the EMT program via mediating EMT-related phenotypes and blocking the Smad2/3 signaling pathway, as well as SBPW3 significantly suppressed the metastatic dissemination of the primary tumor to the liver of mice of CRC metastasis model (Li et al., 2019a).

#### Shenling Baizhu San

Shenling Baizhu San (SBS) is a canonical and popular TCM classical formulation first described in “The Prescriptions of the Bureau of Taiping People’s Welfare Pharmacy” in the Song dynasty and recorded in the Chinese Pharmacopoeia 2020 edition (Yang et al., 2018). Notably, SBS was extensively reported to be competent in mediating the immune response to inflammation, treating diarrhea, and potentiating gastric motility (Ma Q. et al., 2019; Zhang S. et al., 2020), and thus it has been generally used to treat ulcerative colitis and related gastrointestinal disorders in China for a long time. Interestingly, It was found that SBS is effective in attenuating the major symptoms of post operational CRC, including exhaustion and fatigue, nausea, diarrhea, and abdominal distension (Lin X. et al., 2015).

Of note, there is a study revealed that SBS markedly alleviated colitis-associated CRC through inhibition of EMT induced by TGF-β1 signaling, shown as down-regulating Snail, N-cadherin, fibronectin, and vimentin, whereas up-regulating E-cadherin expression. In addition to SBS treatment decreased mortality by reducing the incidence and multiplicity of colonic tumors in azoxymethane/dextran sodium sulfate-induced caCRC mice (Lin X. et al., 2015).

#### Pien-Tze-Huang

Pien-Tze-Huang (PZH) is a well-known Chinese patent medicine formulated by a royal physician in the Ming dynasty. Of note, it has been categorized as one of the national treasures in the catalogue of the National Protected Traditional Chinese Medicines. Fascinatingly, it has been described to have traditional benefits of heat-clearing, detoxification, facilitation of blood circulation, and removal of blood stasis. Recently, the toxic dampness and heat are the primary causes for committing the pathogenesis of cancers in the traditional Chinese medicines theory, and PZH has long been used as an alternative anti-cancer agent for cancers treatment in China and Southeast Asia.

In recent years, it has attracted a lot of attention attribute to be effective in the treatment of CRC in terms of inhibiting tumor cell proliferation, suppressing metastatic and invasive potential, as well as repressing EMT progression (Shen et al., 2012; Zhuang et al., 2012; Lin W. et al., 2015). Notably, PZH has exhibited therapeutic efficacy against tumor metastasis and EMT by targeting the TGF-β/Smads signaling pathway, resulting in a decrease in N-cadherin, TGF-β, p-Smad2/3, and Smad4 expressions, while an increase in E-cadherin expression *in vitro* and *in vivo* (Lin W. et al., 2015). Similarly, PZH has also been shown to effectively suppress EMT process via EMT-related morphological changes and mediation of TGF-β signaling pathway activation in HCT-8/5-fluorouracil (5-Fu) cells (Shen et al., 2014). Likewise, PZH could remarkably restrain the metastatic and invasive capability of CRC cells by reversing the EMT process by down-regulating the key mediators of TGF-β1 signaling in CRC HCT-8 cells (Shen et al., 2015).

In summary, these accumulating pharmacological investigations demonstrated that TGF-β1/Smad plays a crucial role in the regulation of metastatic CRC, and these above-mentioned Chinese herbal compounds and formulas exerted strong inhibitory activities on the invasion and metastasis potential of CRC via down-regulating EMT-related TGF-β/Smad signal pathway.

#### PI3K/Akt Signaling in EMT

The PI3K/Akt signaling, as a Smad-independent pathway also positively participates in the TGF-β molecular pathway induced EMT program (Zhang, 2017a; Hao et al., 2019), which can promote the activation of the mTOR complex and NF-κB, eventually resulting in facilitating EMT-TFs Snail and Slug expressions. On the other hand, glycogen synthase kinase 3 beta (GSK-3β) is a downstream regulator of the PI3K/AKT signaling, its activation phosphorylated the 9th residue of GSK-3β, facilitating GSK-3β ubiquitination and subsequent degradation, and thus, GSK-3β can not involve in the degradation of Snail (Xu et al., 2015). Moreover, its degradation after the PI3K/Akt signaling activation can directly increase the nuclear translocation of β-catenin and intracellular expressions of Snail and Slug in Smad-independent pathways, leading to the promotion of the EMT process. Collectively, the PI3K/Akt signaling is a potential target positively involved in the EMT process and has attracted intense attention for the treatment of CRC metastasis.

#### Scutellaria Barbata D.Don [Lamiaceae] Ethanol Extract

It has been reported that the SBEE can promote apoptosis and repress cell proliferation in colon cancer cells (Lin J. et al., 2014; Wei et al., 2017). SBEE has revealed a remarkable suppressive efficacy on activating the PI3K/AKT signal pathway in CRC HCT-8/5-Fu cells (Lin J. et al., 2017). Likewise, one prior study illustrated that SBEE in a dose-dependent manner markedly decreased the migratory and invasive ability of HCT-8 cells by inhibiting the activation of PI3K/AKT signalings (Jin et al., 2017).

#### Jiedu Sangen Decoction

The Jiedu Sangen Decoction (JSD) is a famous Chinese herbal prescription composed of three natural medicinal herbs (Yuan et al., 2019c). It has been extensively used to treat gastrointestinal cancer, especially as an effective adjuvant in the therapy of CRC patients manifested as ameliorating clinical symptoms and improving living quality. Of note, JSD has been revealed to be beneficial in decreasing the migratory and invasive potential of CRC CT-26 cells (Shan-Ming et al., 2013).

The PI3K/AKT pathway is essential in regulating EMT-associated genes expressions, and JSD has presented as exhibiting a remarkable inhibitory effect on tumor growth via suppressing PI3K/AK signaling pathway in HCT-8/5-Fu cells and in tumor xenograft mice model (Sun et al., 2021). Furtherly, it has been published that JSD combined with PD-L1 inhibitor markedly repressed the metastasis and invasive ability of CT-26 cells *in vitro* and *in vivo* by reversing the EMT process via modulation of the PI3K/AKT signaling pathway (Shan et al., 2020). Interestingly, JSD could significantly reverse EMT progression and inhibit the invasion and metastasis potential of SW480 and SW620 cell lines via suppression of the AKT/GSK-3β signaling pathway activation (Yuan et al., 2019a). There is another study confirmed that JSD notably repressed the migration and invasion of SW480 cells and reversed the EMT status through mediating the EMT-associated genes containing the E-Cadherin, N-Cadherin, and transcriptional coactivator with the PDZ-binding motif expressions in both the SW480 cells and tumor tissues of liver metastasis colon cancer model mice (Yuan et al., 2019c).

#### Resveratrol

Accumulating evidence has confirmed that resveratrol was well in modulating migration and invasion of tumor cells by reversion of EMT regulation in glioma and pancreatic cancer (Li et al., 2013; Cilibrasi et al., 2017). Interestingly, it was displayed that treatment with resveratrol was also conducive to reducing the tumor invasion and metastasis capabilities in AKT1-knockdown SW480 and SW620 cells, and remarkably up-regulated E-cadherin expression and down-regulated p-AKT1, p-GSK-3β, Snail, and N-cadherin expressions in the CRC cells and in a lung metastasis mice model inoculated with SW480 cells. Collectively, resveratrol may be a potential EMT-reversal agent for the treatment of metastatic CRC by restraining the AKT/GSK-3β/Snail signal pathway (Yuan et al., 2019b).

### NF-κB Signaling in EMT

NF-κB is a crucial EMT-inducing transcription factor, and it is shown to prevent EMT-induced metastasis through attenuating NF-κB signaling in CRC cell systems (Liang and Huang, 2016; Ma et al., 2016). It is documented that the PI3K/AKT signaling can cooperate with the TGF-β pathway, which in turn triggers the activation of the mTOR complex and NF-κB, synergistically promoting the EMT program (Dongre and Weinberg, 2019).

#### Resveratrol

Interestingly, there is another study also demonstrated that resveratrol is obviously competent at suppressing TNF-β-induced activation of tumor-promoting transcription factor NF-κB, and thus mediating EMT signatures containing Slug, E-cadherin, and vimentin, resulting in exerting inhibitory effects of EMT program in CRC HCT116 and HCT116R cell lines ( Buhrmann et al., 2018 ). Analogously, treatment with resveratrol (Buhrmann et al., 2019) dramatically repressed TNF-β-induced NF-κB activation and EMT-associated with the parameters expression including decreasing vimentin and Slug, elevating E-cadherin, consequently leading to inhibit invasion and migration of CRC cells (HCT116, RKO, and SW480). Another study reported that resveratrol suppressed the invasive ability of HCT-116R and SW480R cells via inhibiting EMT indicators vimentin and Slug, elevating E-cadherin and claudin-2 expressions, and down-regulating NF-κB signaling activation (Buhrmann et al., 2015).

#### Apigenin

Apigenin is a common flavonoid found in many fruits, vegetables, and Chinese medicinal herbs, which presents various biological activities including antioxidant, anti-inflammatory, antibacterial, and antiviral activities (Yan et al., 2017). Moreover, apigenin has exerted anti-proliferation and pro-apoptosis effects in different types of cancers, including prostate, ovarian, bladder, gastrointestinal, and colon (Pandey et al., 2012; Tang et al., 2017; Chien et al., 2019). It has been reported that the apigenin strongly repressed the NF-κB/Snail signaling activation, and regulated the expression of EMT-related markers containing E-cadherin, vimentin, N-cadherin, and occludin, thereby resulting in inhibiting the EMT process to invasion and metastasis of human HCT-116 and LOVO cells and in HCT-116 cells-induced xenograft nude mice model (Tong et al., 2019).

#### Shaoyao Decoction

Shaoyao decoction (SYD) is a canonical Chinese medicine prescription formulated by Liu Wan-Su in the Jin-Yuan dynasty, which possesses clearing heat and removing dampness, eliminating stasis and toxin in the intestinal tissues (Xia et al., 2018). Previously, SYD was found to promote mucous cell proliferation, reduce exfoliated mucosal cell proliferation, prevent lamina propria edema, and suppress inflammation response (Lin X. et al., 2014). Hence, SYD has been widely used to prevent and treat diverse diseases associated with damp-heat syndrome in the intestines, including ulcerative colitis and Crohn’s disease (Chen, 2014).

Interestingly, a recent pharmacological study revealed the action of SYD effectively against the development of colitis-associated CRC by suppressing inflammation and oxidative damage *in vivo* (Wang et al., 2019; Wang X. et al., 2020). Of note, SYD has been confirmed to be effective in reversing the EMT program by inhibiting NF-κB p65 activation, thus presented as up-regulation of E-cadherin and down-regulation of N-cadherin, vimentin, fibronectin, and EMT-TFs Snail expressions in the azoxymethane and dextran sodium sulfate-induced colitis-associated CRC model mice and in SW480 and HCT116 cells (Lin X. et al., 2014).

In general, these findings have indicated that targeting PI3K/Akt and associated NF-κB signaling pathways exert inhibitory efficacies on the invasion and metastasis abilities by above TCM, which may be a promising therapeutic strategy.

### Matrix Metalloproteinases in EMT

The extracellular matrix (ECM) plays a pivotal role in the mediation of the tissue microenvironment and the maintenance of cellular homeostasis. Importantly, matrix metalloproteinases (MMPs) are a family of ECM-degrading peptidase mainly associated with the degradation and reconstruction of the ECM, which is positively contributed to tumor invasive and metastatic processes (Cho et al., 2020). Among them, convincing evidence illustrated that the matrix metalloproteinase-2 (MMP-2) and matrix metalloproteinase-9 (MMP-9) are particularly effective in inducing decomposition of ECM and degrading collagen, fibronectin, and elastin which are closely related to metastasis and invasion of tumor cells, thereby facilitating migration and invasion in CRC progression (Gialeli et al., 2011). Importantly, it has been revealed that MMP-2 expression is positively controlled by Smad2 whilst MMP-9 is modulated via NF-κB p65/p50 transcription signaling in tumor tissues and cells of CRC (Muscella et al., 2020; Napoli et al., 2020).

#### Quercetin

Quercetin is a kind of flavonoid compound that originates from natural plants, with high potential in oncology due to its chemopreventive efficacies confirmed *in vitro* and *in vivo* investigations. The activity of MMP-2 and MMP-9 is strongly associated with that of their specific inhibitors, tissue inhibitor of metalloproteinases (TIMPs). Notably, it has been demonstrated that quercetin markedly down-regulated MMP-2 and MMP-9 activities in a dose-dependent manner, whereas significantly increased TIMP-2 and TIMP-1 mRNA expression, and thus effectively exerted the anti-metastatic and anti-invasive effects by modulating the EMT programme in CT-26 cells. In addition, it also markedly decreased lung metastasis of CT-26 cells in an experimental metastasis mice model (Kee et al., 2016).

#### Ginsenoside Rd and 20(R)-Ginsenoside Rg3

Ginsenoside Rd is the bioactive constituent in ginseng, which is widely used to treat cardiovascular diseases, inflammation, and trauma (Tian YZ. et al., 2020). Recently, accumulating evidence shows that ginsenoside Rd has remarkable effects of anti-inflammation, anti-proliferation, pro-apoptosis, and modulating gut flora on several human cancers including breast, liver, gastric cancer, and CRC *in vitro* researches (Huang et al., 2017; Tian YZ. et al., 2020). It has been published that ginsenoside Rd led to a significant decrease of EMT markers containing Snail, Twist, fibronectin, and N-cadherin RNA levels, as well as it displayed a significant lowering effect on MMP-2 and MMP-9 activities, resulting in repressing EMT process in HT29 and SW620 cell lines. Moreover, it reduced the number and size of tumor metastasis nodules in the livers, lungs, and kidneys in the colorectal metastasis mice model (Phi et al., 2019a).

20(R)-Ginsenoside Rg3 (ginsenoside Rg3R) is a natural protopanaxadiol-type ginsenoside component present in ginseng that has been indicated to present multiple biological active properties containing anti-tumor, anti-angiogenesis, and anti-inflammation (Lee et al., 2016; Zhang S. et al., 2017; Nakhjavani et al., 2020). One prior study revealed that ginsenoside Rg3R was conducive to repressing the TGF β1-induced EMT progression, invasive ability, and lung cancer migration (Kim et al., 2014). Similarly, it was also reported that ginsenoside Rg3R treatment notably restrained the migration and invasion abilities of HT29 and SW620 cells, which were mediated by the Snail signaling axis, mainly involved in significant regulation of EMT-associated markers including Snail, EGFR, fibronectin, E-cadherin, and MMP-2 proteins (Phi et al., 2019b).

#### *Rhus chinensis* Mill. [Anacardiaceae] Water Extract

*Rhus chinensis* Mill. [Anacardiaceae] is a commonly used medicinal herb in East Asia. It has been described to exhibit several pharmacological activities, including anti-inflammatory, anti-oxidative, anti-bacterial, and anti-tumor characteristics (Yim et al., 2016; Park et al., 2019). In addition, previous studies have reported that some bioactive compounds were isolated from *Rhus chinensis* Mill. [Anacardiaceae] could restrain proliferation, repress migratory, and promote apoptosis in cancers (Kawk et al., 2018; Liu et al., 2019; Kang et al., 2021).

A recent publication demonstrated that *Rhus chinensis* Mill. [Anacardiaceae] water extract (RCWE) was strongly effective in restraining metastatic and invasive characteristics of CRC cells by modulating EMT-related phenotypes, and suppressed MMP-2 and MMP-9 activities, as well as it also reduced the metastatic tumor nodules in the lungs of CRC lung metastatic model mice (Mun et al., 2019).

#### Oxymatrine

It has elucidated that treatment with oxymatrine was obviously competent at inhibiting the nuclear translocation of the NF-κB p65 (Lu et al., 2015; Halim et al., 2019), and thus it is postulated that it may be efficient in reversing the EMT process regarded as an anti-metastatic agent. Surprisingly, there is a pharmacological investigation revealed that oxymatrine markedly blocked the migration and invasion of CRC RKO cells via inhibiting NF-κB p65 activation, and then regulated the EMT-associated genes, such as E-cadherin, N-cadherin, and Snail expressions to reverse the EMT process (Liang and Huang, 2016). Similarly, It was also found that oxymatrine in combination with 5-Fu treatment efficiently regulated tumor cells EMT through effective modulation of the expressions of Snai2, vimentin, E-cadherin, and p-NF-κB p65 in HCT-8/5-Fu cells (Liang et al., 2020).

#### Berberine

In particular, MMPs are closely modulated by a class of endogenous enzymes, named TIMPs. Among them, TIMP1 with a marked anti-apoptotic efficacy is positively conducive to stimulating cell growth and triggering migration and invasion (Bockelman et al., 2018). Furtherly, TIMP1 levels in serum and tissue are increased in patients with colon cancer. Berberine is a natural compound extracted from the herbal plant *Coptis chinensis* Franch. [Ranunculaceae], which has shown its potential pharmacological activities for various cancers, such as breast, liver, and colon cancers. Fascinatingly, One study reported that berberine strongly down-regulated the expression of two genes involved in the EMT program containing Snai2 and TIMP1 in colon adenocarcinoma HCA-7 cells (Palmieri et al., 2019).

#### *Arctium lappa* L. [Asteraceae] Water and Ethanol Extracts

*Arctium lappa* L. [Asteraceae] is one of the most popular traditional medicinal plants in Asia, which has shown various characteristics of clearing the heat, detoxification, and cancer (Li Y. et al., 2021). Modern pharmacological studies show that it has anti-inflammatory, anti-oxidant, anti-proliferative, and anti-tumor activities (Wu et al., 2014; Kim et al., 2020). One prior study has proven that *Arctium lappa* L. [Asteraceae] water extract (ALWE) and ethanol extract (ALEE) strongly inhibited the EMT program by up-regulating the epithelial signature, E-cadherin, down-regulating the mesenchymal marker, N-cadherin expression. Additionally, ALWE could constrain the migration and invasion property of CRC CT-26 cells through inhibiting MMP-2 and MMP-9 activities (Han et al., 2017).

#### Ginseng Ethanol Extract

As is well-known, the root of ginseng is one of the most popular herbal medicines. It has been described to have various pharmacological properties of ginseng containing anti-cancer, anti-inflammatory, anti-oxidant, and anti-aging effects (Liu, 2019; Tian M. et al., 2020). The ginseng ethanol extract exhibited marked inhibitory effects on migration and invasion capability of CT-26 and HT-29 cells by restraining EMT-related parameters including, N-cadherin, vimentin, and Snail expressions, while increased E-cadherin, and reduced MMP-2 and MMP-9 protein levels. In addition, it effectively decreased the pulmonary tumor nodules in lung metastasis model mice injected with CT-26 cells, and mediated EMT-related parameters such as Snail, E-cadherin, N-cadherin, vimentin, and MMP-9 mRNA expressions in the metastatic lung tissues (Kee et al., 2018).

In brief, MMP-2 and MMP-9 proteins are two crucial facilitators that are conducive to potentiating tumor metastatic and invasive potentials, these therapeutic candidate agents are expected to be used to the clinic CRC treatment and intervention by targeting the two crucial targets.

#### JianPi JieDu Recipe

JianPi JieDu Recipe (JPJD) is a TCM formula based on clinical treatment experience, with the efficacies of tonifying Qi and spleen, eliminating dampness, and regulating Qi and detoxification. Recent clinical basic research has confirmed the anti-cancer effect of JPJD and has revealed that JPJD efficiently ameliorated patients’ symptoms and improved living quality, elevated therapeutic efficacy, and decreased toxicity for the treatment of CRC (Sui et al., 2014). Interestingly, JPJD has been usually used to protect against gastrointestinal cancer metastasis and recurrence.

A pharmacological study showed that JPJD drastically inhibited the tumor invasive and migratory capability by inhibition of TGF-β/Smad signaling pathway, then modulating the EMT-related signatures containing Snail, E-cadherin, and vimentin expression levels. Moreover, it markedly repressed the liver and lung metastasis, up-regulated the E-cadherin and Smad2/3, and down-regulated the vimentin, p-Smad2/3, and Snail expressions in orthotopic CRC model mice (Liu et al., 2017). Overall, it was considered as a potential alternative TCM formulation to overcome the metastasis and invasion potential of CRC in clinical treatment.

### MiR-200 Family in EMT

The miR-200 family is well known to be regarded as EMT suppressors intimately involved in tumor metastasis, and it consists of five members containing miR-200a, miR-200b, miR-200c, miR-429, and miR-141 (Hur et al., 2013; Feng et al., 2014; Lee YJ. et al., 2018). ZEB1 and ZEB2, which are identified as the most prominent downstream factors of the miR-200 family, act as direct repressors of E-cadherin in EMT regulation. In the upstream signal transduction, the miR-200 family can be hindered by the kinase Akt, and PI3K/AKT signaling pathway is positively involved in repressing its activation. Recent evidence revealed that miRNAs from the miR-200 family could maintain the epithelial phenotype E-cadherin expression by targeting EMT-TFs ZEB1/2 levels, leading to inhibition of cancer cell migration and motility (Kim et al., 2019; Rokavec et al., 2019; Babaei et al., 2021).

#### Curcumin

Curcumin is a bioactive compound separated from the rhizomes of *Curcuma longa* L. [Zingiberaceae] (Kunnumakkara et al., 2017). It has been extensively investigated in preclinical trials and used in the clinical treatment of various cancers, such as pancreatic cancer, breast cancer, and CRC (Bimonte et al., 2016; Tomeh et al., 2019; Mansouri et al., 2020; Pricci et al., 2020). Epilepsy-ataxia syndrome (EPM5) is a direct target of miR-200c and ectopic expression of EPM5 alone is sufficiently able to induce EMT in CRC. Notably, it has been proven that curcumin treatment was effective in inhibiting the EMT process in SW620 and HT29 CRC cells by up-regulation of miR-200c and down-regulation of EPM5, suggesting that curcumin might be able to prevent or restrain tumor metastasis in CRC progression (Wang H. et al., 2020).

#### Resveratrol

Resveratrol is a natural polyphenol that is abundant in grape skin and seeds. Pharmacological reports have shown that resveratrol significantly restrained the migration and invasion capacities of HCT116 cells through up-regulating of miR-200c expression, resulting in a considerable reduction in vimentin and ZEB1 expressions and a remarkable in increase E-cadherin expression by verification of LNA-anti-miR-200c transfection method (Karimi Dermani et al., 2017).

#### Pien-Tze-Huang

PZH is a classical TCM formulation that has also been considered as a folk remedy used in the treatment of various types of human cancer in China and Southeast Asia for centuries. In a vitro study, PZH strongly suppressed the migration and invasion features of HCT-8 cells in a dose-dependent manner, which might be associated with enhancing the miR-200a, miR-200b, and miR-200c expressions, consequently leading to regulation of EMT-related proteins including N-cadherin and E-cadherin (Shen et al., 2015).

Taken together, these researches suggested that curcumin, resveratrol, and PZH may become a potential adjuvant therapeutic candidate used in suppressing metastasis of CRC via enhancing miR-200 family expressions, which further verified through more reliable pharmacological evidence in the future.

### Wnt/β-Catenin Signaling in EMT

Wnt/β-catenin signaling pathway plays a vital role in modulating the EMT programme in CRC. Wnt signals are transduced across the plasma membrane by Frizzled and low-density lipoprotein receptor-related protein (LRP). In the absence of signaling, *ß*-catenin is phosphorylated by a complex of GSK-3β, Axin, and the tumor suppressor adenomatous polyposis coli (APC), which sequesters *ß*-catenin in the cytoplasm and marks it for proteasomal degradation (Fodde and Brabletz, 2007). Activation of Wnt signaling represses the destruction complex containing GSK-3β through disheveled (Du and Shim, 2016) (DSH), then facilitating *ß*-catenin translocates into the nucleus to bind to members of the T-cell factor (TCF)/Lymphoid Enhancing Factor (LEF) family of transcription factors and induce transcription of the Wnt genes. Consequently, Wnt signaling (Gonzalez and Medici, 2014) is inappropriately active and directly triggers EMT-TFs expressions containing Snail1 and Snail2, which strongly promotes the EMT process (Lu et al., 2021).

#### Curcumin

Recently, increasing numbers of studies have found that curcumin plays a vital role in inhibiting the EMT process in CRC (Lee YH. et al., 2018; Su et al., 2018). It was found that curcumin might exert anti-metastatic efficacy via reversing the EMT programme through mediating the Tet1-Nkd2-Wnt signaling in HCT116 cells (Lu et al., 2020). Further, it was also reported that curcumin (Zhang et al., 2016) strongly hindered tumor EMT through downregulating Nkd2-Wnt-CXCR4 signaling, then mediation of EMT-associated markers E-cadherin and vimentin expressions, resulting in a reduction of invasive and metastatic abilities in SW620 CRC cells. In an additional study (Chen T. et al., 2020), curcumin significantly reduced the expression of EMT-related genes containing Wnt3a, Snail1, and Twist, N-cadherin, and vimentin, as well as nuclear translocation levels of *ß*-catenin, while remarkably increasing E-cadherin expression, consequently suppressing EMT progression in SW480 CRC cells.

#### Genistein

Genistein, a beneficial soy-derived isoflavone and is well known for its various biological activities, such as the inhibition of inflammatory, promotion of apoptosis, anti-proliferative, and anti-bacterial effects (Erten et al., 2021). Convincing evidence has shown that genistein can repress the proliferation and induce apoptosis of CRC cells (Shafiee et al., 2016; Liang et al., 2018; Zhang L. et al., 2020).

It was prominent that genistein treatment could obviously repress the migration ability of prostate, melanoma, and mammary cancers cell lines (Mukund et al., 2017). Likewise, it has been generally described to be effective in repressing the proliferation and inducing apoptosis of CRC cells (Shafiee et al., 2016; Liang et al., 2018; Zhang L. et al., 2020). Wnt inhibitory factor 1 (WIF1) is a tumor suppressor, which can interact with Wnt protein to inhibit the Wnt pathways. It has been reported that silencing of WIF1 is positively related to CRC (Sanchez-Vega et al., 2017). Interestingly, genistein obviously repressed the invasive and metastatic abilities of HT29 cells by recovering the activity of WIF1 through altering Wnt-1/β-catenin signaling-associated factors containing *ß*-catenin, c-Myc, and cyclin D1 expressions, and the MMP-2 and MMP-9 expression levels were notably reduced, while the expressions of metalloproteinase inhibitor 1 and E-cadherin were increased drastically (Zhu et al., 2018). Taken together, it may be a promising therapy for the clinical treatment of CRC.

#### Triptolide

Triptolide, a diterpene triepoxide that is a major bioactive natural compound isolated from *Tripterygium wilfordii* Hook.f. [Celastraceae], which is well known for its anti-inflammatory effect (Noel et al., 2019; Li et al., 2020). Recently, triptolide has been demonstrated as a potential anti-tumor agent in pharmacological research regarding CRC therapy. The previous report confirmed that triptolide treatment exerted inhibitory effects on tumor metastasis via blocking the activation of Wnt/β-catenin and NF-κB signalings, subsequently mediating EMT-related indicators expressions containing E-cadherin, vimentin, and N-cadherin in HT-29 and SW480 cells (Qi and Li, 2019). Further, it was found that triptolide was able to in inhibiting CD133^+^/CD44^+^ colon cancer stem cell migration through repressing EMT program, which manifested as reducing the EMT-TFs, such as Snail, Slug, and twist expressions (Acikgoz et al., 2020).

#### Apigenin

Apigenin is a natural plant flavonoid and has widely exhibited anti-cancer efficacies through multiple bioactive effects (Singh et al., 2019), such as inducing cell cycle arrest, triggering cell apoptosis, and anti-metastasis *in vitro* and *in vivo* studies (Zhu et al., 2015; Yammine et al., 2021). Of note, apigenin could markedly suppress *ß*-catenin/TCF/LEF signaling activation, which was induced by LiCl. In addition, it markedly inhibited tumor metastasis and invasion via repressing the activation of Wnt/β-catenin signaling in CRC (Xu et al., 2016; Lin CM. et al., 2017).

Therefore, the suppression of Wnt/β-catenin signaling is also one of the potential approaches to fighting metastatic CRC, suggesting the aforementioned TCMs is effective in suppressing the invasion and metastasis potential of tumor cells and may be contributed to the development of therapeutic agent.

### Notch Signaling in EMT

The Notch signaling is another EMT-activating route, which can be activated by Notch binding to Delta and Jagged ligands (Liu et al., 2018). After activation, the Notch intracellular domain (Notch ICD) is released through a cascade of proteolytic cleavages, which enters the nucleus and triggers CSL (CBF1/Su(H)/Lag-1) transcription factor to express EMT-TFs (Du and Shim, 2016). Notch signaling is effective in promoting Snail1 and Snail2 expressions, which binds to E-boxes in the human E-cadherin promoter and inhibits the E-cadherin expression (Liu et al., 2018; Avila-Carrasco et al., 2019). Furthermore, Notch directly stimulates the Slug promoter and subsequently leads to an increase of Slug expression, thereby resulting in transforming to EMT phenotype (Kar et al., 2019).

#### Genistein

Genistein is the natural bioactive isoflavone with a broad range of pharmacological properties. It has been reported that genistein (Zhou et al., 2017) was effective in inhibiting migration of HT-29 cells through the reversion of the EMT progression via suppressing the Notch1 pathway, which was manifested as mediating EMT-signatures including E-cadherin and N-cadherin, and EMT-TFs, such as Snail2/Slug, ZEB1, ZEB2, FOXC1, FOXC2, and Twist1. Besides, it remarkably inhibited the NF-κB and p-NF-κB expressions.

#### Ginsenoside Rg3

Ginsenoside Rg3 is a bioactive ginseng compound that has been shown to possess anti-metastatic activity in colon cancer. It has been reported that ginsenoside Rg3 could effectively inhibit invasion and metastases of HCT15 cells and SW48 cells through restraining the Notch-Hes1-EMT signal pathway activation, specifically increasing E-cadherin, while decreasing EMT-related markers vimentin, Snail, Notch ICD, and Hes1 expressions, as well as decreasing the tumor metastasis nodules in the liver of metastasis model mice (Li X. et al., 2021).

To sum up, the molecular mechanisms of a large number of bioactive compounds and extracts derived from natural herbal plants and Chinese medicine formulas against metastatic and invasive characteristics of CRC *in vitro* and *in vivo* models are summarized in Tables 1, 2 and 3, respectively. Taken together, these candidates have great promising inhibitory efficacy on the invasion and metastasis of CRC via reversing EMT-related signalings in pharmacological studies.

**TABLE 1:** Targeting of EMT-related signaling pathways involved in metastatic CRC by natural bioactive compounds *in vitro* and *in vivo*.

Compound names	Cell lines/animal models	Effective dose	Mechanisms of action	References
Curcumin	rHCT-116 cells	5, 10, 20, and 40 μmol/L	Inhibit EMT progress; E-cadherin, *ß*-catenin, TCF4 and Axin (↓); TET1, NKD2, and vimentin (↑)	Lu et al. (2020)
	SW620 cells	10, 20, and 40 μmol/L	Inhibit tumor metastasis; vimentin and CXCR4 (↓); E-cadherin, NKD2 (↑)	Zhang et al. (2016)
	SW620 and HT29 cells	/	Repress migration and invasion; EPM5, Snail, Slug, and ZEB1/2, and vimentin (↓); miR-200c, CDH1 (↑)	Wang et al. (2020a)
	SW480 cells	0.1, 0.2, and 0.4 μmol/L	Suppress EMT transform; Wnt3a, Snail1, and Twist	Chen et al. (2020b)
			N-cadherin, and vimentin, *ß*-catenin (↓); E-cadherin (↑)	
Quercetin	CT-26 cells	0.1, 1, and 10 μmol/L	Repress tumor metastasis and invasion; N-cadherin	Kee et al. (2016)
	CT-26 cells-induced lung metastases mice model	10, 50 mg/kg	β-catenin, Snail, MMP-2, and MMP-9 (↓); E-cadherin (↑)	
	SW480 cells	25, 50, and 100 μmol/L	Repress EMT-like phenotypes and tumor metastasis; Twist1 and vimentin (↓); E-cadherin (↑)	Feng et al. (2018)
Resveratrol	HCT116 and HCT116R cells	1, 5, and 10 μmol/L	Restrain the tumor invasion; NF-κB, vimentin, Slug, MMP-9, and caspase-3 (↓); E-cadherin (↑)	Buhrmann et al. (2015)
	HCT116 and HCT116R cells	5 μmol/L	Suppress invasion; NF-κB, MMP-9, CXCR4, vimentin, and Slug (↓); E-cadherin (↑)	Buhrmann et al. (2018)
	HCT116, RKO, and SW480 cells	5 μmol/L	Inhibit tumor migration and invasion; NF-κB, vimentin, and Slug (↓); E-cadherin (↑)	Buhrmann et al. (2019)
	LoVo cells	6, 12 μmol/L	Reduce the rate of lung and hepatic metastases; p-Smad2/3, Snail, Slug, vimentin, MMP-2, and MMP-9 (↓); Smad2/3 and E-cadherin (↑)	Ji et al. (2015)
	LoVo cells-induced lung metastases mice model	50, 100, and 150 mg/kg		
Resveratrol	SW480 and SW620 cells	15 μmol/L	Inhibit CRC cell migration and invasion; N-cadherin, p-AKT1, p-GSK-3β, and Snail (↓); E-cadherin (↑)	Yuan et al. (2019b)
	Epidermal growth factor-induced EMT mice model	150 mg/kg		
	HCT-116 cells	30 μmol/L	Hinder the tumor migration and invasion; vimentin and ZEB1 (↓); miR-200c, E-cadherin (↑)	Karimi Dermani et al. (2017)
Berberine	EMT-like HcoEpiC cells	25, 50, and 100 μg/ml	Inhibit EMT program; TβRI, TβRII, Smad2/p-Smad2, Smad3/p-Smad3, ZEB1, and Snail (↓)	Huang et al. (2020)
	HCA-7 cells	10, 30 μmol/L	Suppress EMT progression; Snai2 and TIMP1 (↓)	Palmieri et al. (2019)
Demethyleneberberine	HCT-116 cells	6, 12, and 18 μmol/L	Promote apoptosis and reverse EMT process; p-Smad2 and p-Smad3 (↓), E-cadherin and ZO-1 (↑)	Wang et al. (2021b)
Ginsenoside Rb2	HCT116 and SW620 cells	0.1, 1, and 10 μg/ml	Inhibit tumor metastasis and EMT program; TGF-β1, Smad4, and p-Smad2/3 (↓)	Dai et al. (2019)
	HT29 and SW620 CRC cells	10, 50, and 100 μmol/L	Decrease tumor metastasis and invasion; Snail, Twist, fibronectin, vimentin, and MMP-2 (↓), E-cadherin (↑)	Phi et al. (2018)
Ginsenoside Rd	HT29 and SW620 cells	10, 50, and 100 μmol/L	Reduce the migration, invasion, and wound-healing abilities; Snail, Twist, fibronectin, N-Cadherin, and p-EGFR (↓); EGFR (↑)	Phi et al. (2019a)
Ginsenoside Rg3	HCT15 and SW48 cells	100 μmol/L	Inhibit tumor invasion and metastasis; vimentin, Snail, NICD, and Hes1 (↓); E-cadherin (↑)	Li et al. (2021a)
	HCT15 cells-induced metastasis mice model	10 mg/kg		
Ginsenoside Rg3R	HT29 and SW620 cells	10, 50, and 100 μmol/L	Inhibit tumor migration and invasion; EGFR, fibronectin, Snail, and MMP-2 (↓), E-cadherin (↑)	Phi et al. (2019b)
	HT29 cells-induced metastases mice model	5 mg/kg		
Triptolide	HT-29 and SW480 CRC cells	10, 50, and 100 nmol/L	Reduce migratory capacity and repress the growth of primary tumor xenografts; Snail, vimentin, and N-cadherin (↓)	Qi and Li, (2019)
			E-cadherin (↑)	
	CD133+/CD44 + colon cancer stem cells	25, 50, 75, and 100 nmol/L	Suppressed migration and EMT processes; Snail, Slug, and Twist (↓)	Acikgoz et al. (2020)
Oxymatrine	RKO cells	0.25, 0.5, and 0.75 mg/ml	Hinder the CRC cells migration and invasion; N-cadherin, Snail and NF-κB p65 (↓); E-cadherin (↑)	Liang and Huang, (2016)
	HCT-8 and HCT-8/5-Fu cells	2 mg/ml	Reverse EMT program, Snai2, vimentin, and p-NF-κB p65 (↓), E-cadherin (↑)	Liang et al. (2020)
	RKO cells	0.25, 0.5 mg/ml	Inhibit the migration of CRC cells; PAI-1, TGF-β1, α-SMA, FN, Smad4, p-Smad2, and p-P38 (↓); E-cadherin (↑)	Wang et al. (2017b)
Apigenin	HCT-116 and LOVO cells xenograft model nude mice	10, 20 μmol/L	Inhibited tumor metastasis, invasion; NF-κB, Snail, N-cadherin, and vimentin (↓); E-cadherin and occludin (↑)	Tong et al. (2019)
		200, 300 mg/kg		
Genistein	HT-29 cells	200 μmol/L	Restrain the migratory capability; N-cadherin, Snail2/Slug, ZEB1, ZEB2, FOXC1, FOXC2 and Twist1 (↓); E-cadherin (↑)	Zhou et al. (2017)
	HT-29 cells	10, 20, and 60 μmol/L	Repress the invasive and metastatic abilities; *ß*-catenin, c-Myc, cyclin D1, MMP-2, and MMP-9 (↓); WIF1, metalloproteinase inhibitor 1, and E-cadherin (↑)	Zhu et al. (2018)

**TABLE 2:** Targeting of EMT-related signaling pathways involved in metastatic CRC by medicinal herb extracts *in vitro* and *in vivo*.

Natural herb extracts or polysaccharide	Extraction procedure	Cell lines/animal models	Effective dose	Molecular mechanism	References
*Scutellaria barbata* D.Don [Lamiaceae] ethanol extract	500 g of *Scutellaria barbata* D.Don [Lamiaceae] was extracted with 5,000 ml of 85% ethanol using a refluxing method and were filtered, and then the ethanol solvent was evaporated and concentrated to a relative density of 1.05	HCT-8 cells	0.125, 0.25, and 0.5 mg/ml	Repress migration and invasion of HCT-8 cells; p-PI3K, PI3K, p-AKT, TGF-β, Smad2/3, Smad4, MMP-1, MMP-2, MMP-3/10, MMP-9, MMP-13 (↓)	Jin et al. (2017)
SBPW3	The crude polysaccharide from *Scutellaria barbata* D.Don [Lamiaceae] was dissolved in distilled water and filtered. After the filtering solution was loaded onto a DEAE cellulose column, the column was eluted with a stepwise gradient of NaCl solutions, and the eluents were collected using an automatic collector. The distilled water eluted fraction was obtained and further purified on a Superdex 200 gel filtration chromatography column with NaCl to yield one fraction, SBPW3	HT-29 cells	50, 100, and 200 μg/ml	Inhibit the tumor migration and invasion	Li et al. (2019a)
		CRC metastasis mice model	100, 200 mg/kg	α-SMA, N-cadherin, p-Smad2, and p-Smad3 (↓); E-cadherin (↑)	
*Rhus chinensis* Mill. [Anacardiaceae] water extract	*Rhus chinensis* Mill. [Anacardiaceae] (100 g) was boiled at 100°C for 3 h with 1 L of distilled water, then the aqueous extract was filtered through whatman filter paper and lyophilized	CT-26 cells colorectal lung metastasis mice model	1, 5, and 10 μg/ml	Repress the migratory and invasive abilities and reverse EMT program	Mun et al. (2019)
			250, 500 mg/kg	N-cadherin, vimentin, Twist, MMP-2 and MMP-9 (↓); E-cadherin (↑)	
*Arctium lappa* L. [Asteraceae] water extract and ethanol extracts	***Arctium lappa* L. [Asteraceae**] **water extract:** The water-soluble components of the *Arctium lappa* L. [Asteraceae] powder were extracted with water by heating at 100°C for 3 h, and boiled solution was filtered and the filtrates were lyoph-ilized, thus the resulting powder was used as the crude total extract of the dried fruit	CT-26 cell lines	1, 10, and 100 μg/ml	Reduce the tumor migration and invasion ability; N-cadherin, MMP-2, and MMP-9 (↓), E-cadherin (↑)	Han et al. (2017)
	***Arctium lappa* L. [Asteraceae**] **ethanol extract**				
	The *Arctium lappa* L. [Asteraceae] (100 g) was extracted with 70% ethanol for 3 h using a heating mantle. The solvents were filtered and evaporated under reduced pressure, and the remnant was then freeze dried at -56°C to acquire extracts of the herbal sample				
Ginseng ethanol extract	100 g of ginseng was decocted in 1 L of ethanol and extracted at 100°C for 3 h. After filtering the ethanol extraction solution, the ethanol solvent was removed by evaporation, and the remaining liquid was freeze-dried	CT-26 and HT-29 cells	0.2 mg/ml	Inhibit migration and invasion of CRC cells; N-cadherin, vimentin, Snail, MMP-2 and MMP-9 (↓); E-cadherin (↑)	Kee et al. (2018)
		CT-26 cells-induced lung metastasis model mice	50 mg/kg		

**TABLE 3:** Targeting of EMT-related signaling pathways involved in metastatic CRC by Chinese herbal formulas *in vitro* and *in vivo*.

Chinese herbal formulas	Main ingredients and proportion	Extraction procedure	Cell lines/animal models	Effective dose	Molecular mechanism	References
Jiedu Sangen Decoction	*Actinidia arguta* (Siebold and Zucc.) Planch. ex Miq. [Actinidiaceae], *Adina rubella* Hance [Rubiaceae], and *Reynoutria japonica* Houtt. [Polygonaceae]. (the ratio of 1:1:1)	Three medicinal herbs of JSD were mixed and soaked in 1 L of distilled water for 30 min. The filtrates were concentrated to 150 ml to obtain JSD such that its crude drug concentration was 2 g/ml of mother liquor	SW480 cells liver metastasis of colon cancer model mice	6 mg/ml	Inhibit the invasion and metastasis of CRC cells; N-Cadherin, Yes-associated protein, PDZ-binding motif (↓); E-Cadherin (↑)	Yuan et al. (2019c)
				24 g/kg		
			CT-26 cells hepatic metastatic CRC mice model	6 mg/ml	Inhibit tumor migration and invasion; PI3K, AKT, Slug, Snail, N-cadherin, and vimentin (↓); E-cadherin (↑)	Shan et al. (2020)
				24 g/kg		
			SW480 and SW620 cells	6 mg/ml	Repress the invasion and metastasis potential; Snail, Slug, Twist, N-cadherin, vimentin, AKT1, p-AKT1, and p-GSK-3β (↓); E-cadherin (↑)	Yuan et al. (2019a)
			SW480 cells-induced liver metastasis model mice	24 g/kg		
JianPi JieDu Recipe	*Astragalus mongholicus* Bunge [Fabaceae], *Atractylodes macrocephala* Koidz. [Asteraceae], *Vitis heyneana* Schult. [Vitaceae], *Akebia quinata* (Thunb. ex Houtt.) Decne. [Lardizabalaceae], *Salvia chinensis* Benth. [Lamiaceae], and *Tetradium ruticarpum* (A.Juss.) T.G.Hartley [Rutaceae]. (the ratio of 10:5:5:8:10:10:10:8)	The herb mixtures of JPJD were heated to 100°C for 3 h, and then the decoction was filter, thus aqueous solution was prepared	LoVo cells	12.5, 25, and 50 μg/ml	Inhibit the invasive and metastatic ability; vimentin, p-Smad2/3, and Snail (↓); Smad2/3 and E-cadherin (↑)	Liu et al. (2017)
			LoVo cells-induced orthotopic CRC mice model	250, 500, and 1000 mg/kg		
Shaoyao decoction	*Areca catechu* L. [Arecaceae], Rheum palmatum L. [Polygonaceae], *Angelica sinensis* (Oliv.) Diels [Apiaceae], *Glycyrrhiza uralensis* Fisch. ex DC. [Fabaceae], *Paeonia lactiflora* Pall. [Paeoniaceae], *Dolomiaea costus* (Falc.) Kasana and A.K.Pandey [Asteraceae], *Cinnamomum aromaticum* Nees [Lauraceae], *Coptis chinensis* Franch. [Ranunculaceae], and *Scutellaria baicalensis* Georgi [Lamiaceae]. (the ratio of 30:15:15:6:6:6:9:15:7.5)	Aqueous extracts of Shaoyao decoction were heated in 10 volumes of distilled water (*v/m*) at 80°C by stirring for 1 h. The extracts were centrifuged at 1, 500 g, and then gained the Shaoyao decoction supernatant, and subjected to condensation under reduced pressure at 70°C	SW480 and HCT116 cells	2, 4 mg/ml	Block EMT process and attenuate proinflammatory cytokines	Lin et al. (2014b)
			AOM/DSS-induced caCRC mice model	7.12 g/kg	Snail, N-cadherin, fibronectin, and vimentin, as well as IL-1β, IL-6, TNF-α, and NF-κB p65 (↓); E-cadherin (↑)	
Shenling Baizhu San	*Panax ginseng* C.A.Mey. [Araliaceae], *Smilax glabra* Roxb. [Smilacaceae], *Nelumbo nucifera* Gaertn. [Nelumbonaceae], *Lablab purpureus* subsp. purpureus [Fabaceae], *Coix lacryma-jobi* L. [Poaceae], *Dioscorea oppositifolia* L. [Dioscoreaceae]	The medicinal medicines of SBS were decocted at 80°C for 1 h using 10 volumes of distilled water (*v/m*), then centrifuged at 1, 500 × g, thus the supernatant was obtained and subjected to reduce pressure at 70°C	AOM/DSS-induced caCRC mice model	7.28 g/kg	Inhibit TGF-β1-induced EMT program	Lin et al. (2015b)
	*Atractylodes macrocephala* Koidz. [Asteraceae], *Platycodon grandiflorus* (Jacq.) A.DC. [Campanulaceae], *Wurfbainia villosa* (Lour.) Skornick. and A.D.Poulsen [Zingiberaceae], and *Glycyrrhiza uralensis* Fisch. ex DC. [Fabaceae]. (the ratio of 15:15:15:12:15:9:9:6:9:6)				Snail, N-cadherin, fibronectin, and vimentin (↓); E-cadherin (↑)	
Pien-Tze-Huang	*Panax notoginseng* (Burkill) F.H.Chen [Araliaceae], musk, Calculus Bovis, and snake gall. (the percentage of 85, 3, 5, and 7%)	PZH was obtained from, and authenticated by the sole manufacturer Zhangzhou Pien Tze Huang Pharmaceutical Co. Ltd.	CT-26 cells	0.25 and 0.5 mg/ml	Hinder the migration and invasion of CT-26 cells and inhibit liver metastasis	Lin et al. (2015a)
			CT-26 cells-induced orthotopic CRC mice model	234 mg/kg	N-cadherin, TGF-β, p-Smad2/3, and Smad4 (↓); E-cadherin (↑)	
			HCT-8/5-Fu Cells	0.25, 0.50, and 0.75 mg/ml	Inhibit the migratory and invasive capabilities of HCT-8 cells; TGF-β, Smad4, ZEB1, ZEB2, and N-cadherin (↓)	Shen et al. (2014)
					E-cadherin (↑)	
			HCT-8 cells	0.25, 0.50, and 0.75 mg/ml	Repress the tumor migration and invasion; TGF-β1, Smad2/3, Smad4, ZEB1, ZEB2, and N-cadherin (↓); miR-200a, miR-200b, miR-200c, and E-cadherin (↑)	Shen et al. (2015)

## Conclusion and Prospect

CRC is a disease characterized by the abnormal proliferation of colon and rectal cells. Despite early detection and therapeutic advances in recent decades, the lymphatic and distant metastases remain the main cause of mortality in CRC patients, and the overall survival of advanced CRC remains unsatisfactory (Huang et al., 2018). Tumor metastasis is a critical marker of cancer deterioration, and accumulating evidence suggested that distant metastatic potential is the primary cause of CRC-associated high mortality (Engstrand et al., 2018; Rahbari et al., 2019).

It has been confirmed that EMT transform is closely associated with tumor metastasis (Pastushenko and Blanpain, 2019; Kong MY. et al., 2020). In the context of CRC, EMT transform confers on tumor cells elevated tumor-initiating, invasive, and metastatic capabilities contributing to disseminating into the surrounding stroma, and they can escape the surveillance of the immune system, subsequently, entering into blood vessels or lymph tube and migrating to distant organs. Collectively, EMT transform is regarded as the main contributor of the metastatic shift in CRC (Dongre and Weinberg, 2019; Park et al., 2021), and the complexity EMT program is quite needed to synergistically intervene through multiple layers of regulation.

Recently, increasing numbers of investigations showed that TCMs in combination with western medicine therapy has been shown to have great clinical value and promising therapeutic efficacies involved in decreasing the recurrence and metastasis rate and improving the quality of life in CRC treatment (Fu et al., 2020; Liu et al., 2020; Sun et al., 2020). As a treasure of China, TCMs reversers with properties of multi-target and multi-link as targeted therapeutic agents for EMT-induced progression of metastatic CRC show great potential and have become an inevitable trend.

**FIGURE 2:**
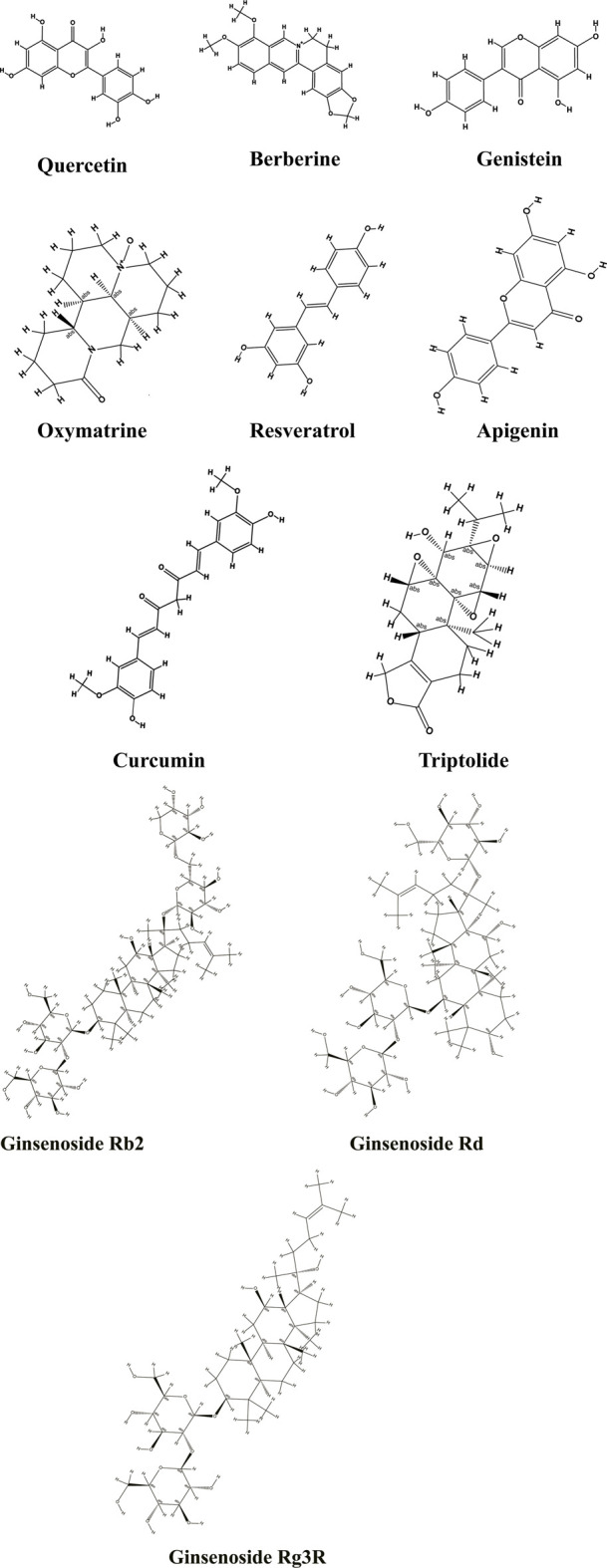
Chemical structure of the main TCMs active constituents with anti-metastatic CRC efficacy.

Research of the anti-metastatic effect of a large number of TCMs monomer compounds and Chinese medicine formulations based on TCM theory or coming from clinical experience have made considerable progress in recent decades. In this article, we mostly focus on certain bioactive compounds, natural herb extracts, and Chinese herbal recipes (Liu et al., 2018; Dai et al., 2019; Wang H. et al., 2020; Guo et al., 2021) repress the EMT program following their specific characteristics to inhibit the invasion and metastasis of CRC. Among them, there is a high proportion of investigations concentrated on TCMs-originated monomers and compounds, while a small number of researches reported Chinese medicine formulas or medicinal herb extracts, as specifically shown in Tables 1–3, moreover, the chemical structure of the main TCMs active compounds with anti-metastatic and anti-invasive efficacies were exhibited in Figure 2. Importantly, there are multiple signal pathways with a large number of interacting constituents and crosstalk molecular mechanisms in EMT transform, and most of the current literature reports mainly concentrate on the classical mechanism of TGF-β/Smads, PI3K/Akt, NF-κB, and Wnt/β-catenin, Notch signaling pathways (Figure 1). Interestingly, we can observe that Chinese herbal prescription including PZH, SBS, JPJD, and natural herb compounds containing resveratrol, berberine, ginsenoside Rb2, oxymatrine, quercetin, and SBPW3 are shown to be obviously effective in combating EMT-induced metastasis of CRC cells via inhibiting TGF-β1/Smads signaling *in vitro* and *in vivo* models experiment. Likewise, three natural plant compounds containing triptolide, curcumin, apigenin are effectively able to regulate Wnt/β-catenin pathway via hindering *ß*-catenin translocates into the nucleus, and resveratrol, SBEE, and JSD exert anti-metastatic effect via the mediation of PI3K/AKT signaling. In addition, genistein and ginsenoside Rg3 are found to constrain the activation of Notch/EMT signaling, accompanied by inhibition of EMT-TFs, such as Snail2/Slug, ZEB1/2, and Twist1, and then modulation of EMT-related indicators expressions, eventually leading to restraining the invasion and metastasis induced by EMT transformation. Furthermore, the therapeutic efficacy of oxymatrine, SYD, resveratrol, and apigenin on attenuating metastatic CRC can be mainly attributed to block NF-κB p65 translocating into the nucleus to repress its activation, resulting in repressing EMT-TFs Snail and Slug.

Furtherly, the RCWE, ALWE, ALEE, ginsenoside Rg3R, and ginseng ethanol extract also exhibit strong inhibitory efficacy on metastasis and invasion potential by restraining MMP-2 and MMP-9 activities, which is effectively conducive to decrease degradation of ECM, thereby inhibiting cell invasion and migration in CRC progression. In addition, PZH and curcumin were demonstrated to repress the EMT programme by up-regulation of miR-200, consequently leading to a reduction of EMT-TFs including ZEB1 and ZEB2 expressions, and thus restraining EMT-associated parameters facilitated to exert the inhibitory efficacy on the metastatic ability of CRC.

However, some of TCMs bioactive components, including quercetin, curcumin, berberine, ginsenoside Rb2, triptolide, oxymatrine, and genistein primarily exhibit their characteristics regarding the anti-metastatic effect via intervening these mentioned signal pathway positively involved in EMT transform in experiments of cell and animal models, while these studies do not truly reflect the pharmacodynamics and pharmacokinetic characteristics of the above-mentioned TCMs in human patients, and there is a lack of the effectiveness evidence of TCMs as anti-metastatic CRC therapeutic agents, ultimately resulting in clinical translation is poor. Furthermore, although a considerable number of traditional herbal prescriptions have been used in clinical treatment and management of other diseases not main CRC for centuries, the specific mechanisms of inhibition migration of CRC under treatment with these herbal recipes are not clearly known. Thereby, it is particularly important to further evaluate whether TCMs can effectively fight against metastatic CRC by authentic preclinical studies and scientific clinical trials to apply for these TCMs in a more safe and reasonable usage, then expectantly finding more potential therapeutic candidates to combat the metastasis of CRC.

Taken together, Chinese herbal medicines with unique advantages of multi-target, multi-paths, and multi-action have been increasingly practiced in fighting against CRC, reducing local invasion and distant dissemination that have attracted much more concern among the pharmacological investigations and preclinical research communities in the past few decades. Therefore, in this direction, these above-mentioned TCMs with anti-metastatic properties may be the great potential and promising therapeutic candidates to control the development and progression of metastatic CRC, which can be considered as an abundant resource of lead compounds for exploring and excavating innovative adjuvant therapeutic agents (Mahadevappa and Kwok, 2017).

In conclusion, EMT plays a key role in the invasion and metastasis of CRC cells; targeting the EMT-related signaling pathways is regarded as an effective and interesting weapon to combat the progression of metastatic CRC. TCM-originated ingredients and Chinese medicine formulas may be effective adjuvant therapeutic agents synergizing with chemotherapeutic drugs, which aid in the clinical management and treatment of metastatic CRC.

## Glossary

EMT: epithelial-mesenchymal transition
CRC: colorectal cancer
TCM: Traditional Chinese Medicine
EMT-TFs: EMT-transcription factors
E-cad: E-cadherin; N-cad, N-cadherin
TGF-β: transforming growth factor-beta
NF-κB: Nuclear factor-κB
PI3K: phosphatidylinositol 3-kinase
AKT (PKB): protein kinase B
ZEB1/2: Zinc-finger E-box-binding 1/2
GSK-3β: glycogen synthase kinase-3 beta
LRP: low density lipoprotein receptor-related protein
APC: adenomatous polyposis coli
DSH: disheveled
TCF: T-cell factor
LEF: lymphoid enhancing factor
Notch ICD: Notch intracellular domain;
5-Fu: 5-fluorouracil
MMP-2: matrix metalloproteinase-2
MMP-9: matrix metalloproteinase-9
TIMP1: tissue inhibitor of metalloproteinase-1
EGFR: epidermal growth factor receptor
EPM5: epilepsy-ataxia syndrome
WIF1: Wnt inhibitory factor 1
Ginseng: Panax ginseng C.A.Mey. [Araliaceae]
SBEE: Scutellaria barbata D.Don [Lamiaceae] ethanol extract
SBS: Shenling Baizhu San
PZH: Pien-Tze-Huang
JSD: Jiedu Sangen Decoction
SYD: Shaoyao decoction
AOM: azoxymethane
DSS: dextran sodium sulfate
RCWE: Rhus chinensis Mill. [Anacardiaceae] water extract
ALWE: Arctium lappa L. [Asteraceae] water extract
ALEE: Arctium lappa L. [Asteraceae] ethanol extract
JPJD: JianPi JieDu Recipe.
